# Regulation of Template Ionization and Template–Monomer Interactions for Enhanced Molecular Imprinting and Selective Detection of Promethazine

**DOI:** 10.3390/polym18172146

**Published:** 2026-09-02

**Authors:** Yang Xing, Xuan Hao Lin, Sam Fong Yau Li

**Affiliations:** 1 Department of Chemistry, National University of Singapore, 3 Science Drive 3, Singapore 117543, Singapore; e1539101@u.nus.edu; 2 NUS Environmental Research Institute (NERI), #02-01, T-Lab Building (TL), 5A Engineering Drive 1, Singapore 117411, Singapore

**Keywords:** molecularly imprinted polymer (MIP), promethazine, quartz crystal microbalance (QCM), molecular recognition, ionization, template–monomer interactions

## Abstract

A molecularly imprinted polymer (MIP)-based quartz crystal microbalance (QCM) sensor was developed for the selective detection of promethazine (PMZ) through deliberate regulation of template–monomer interactions during the molecular imprinting process. Various functional monomer systems were systematically evaluated to optimize intermolecular recognition, among which the combination of 4-vinylpyridine (4-VP) and 2-acrylamido-2-methylpropanesulfonic acid (AAMPS) exhibited the strongest interaction toward PMZ. Furthermore, protonation of PMZ using hydrochloric acid significantly enhanced electrostatic complexation between the template and functional monomers, leading to improved pre-polymerization organization, more effective imprinting-site formation and high selectivity towards target analytes. The resulting MIP-QCM sensor exhibited a linear response toward PMZ over the concentration range of 50–2000 ppb with a correlation coefficient (R^2^) of 0.99148 and a detection limit of 27.4 ppb. The sensor demonstrated favorable selectivity against structurally related compounds, strong anti-interference performance under highly competitive matrix conditions, and excellent regeneration stability, retaining 97.9% of its original response after ten regeneration cycles. Recovery studies in spiked tap water and carbonated beverage samples yielded recoveries ranging from 94.7% to 112.6%. These results demonstrate that regulating template ionization and template–monomer interactions is an effective strategy for improving imprinting-site quality and molecular recognition performance. The proposed approach provides new insight into the rational design of highly selective MIPs for basic pharmaceutical compounds and offers a promising platform for environmental, pharmaceutical and forensic monitoring applications.

## 1. Introduction

Promethazine hydrochloride (PMZ), a typical phenothiazine derivative, is widely used for the treatment of psychiatric and psychomotor disorders due to its antihistaminic, sedative, and antiemetic properties [1,2]. However, excessive use of PMZ can lead to severe adverse effects, particularly involving the central nervous and cardiovascular systems, and may even result in life-threatening conditions [3,4]. Notably, the co-administration and abuse of PMZ with opioids (e.g., codeine) has become an increasing public health concern, as PMZ can potentiate opioid effects and significantly elevate the risk of respiratory depression and central nervous system complications [5,6]. This combination, commonly known as ‘Purple Drank’ when mixed with carbonated beverages, has emerged as a recreational drug of concern, particularly among young adults [5]. Owing to its toxicity to aquatic organisms and long-term environmental persistence, PMZ has been classified as an environmental hazard under the Globally Harmonized System of Classification and Labelling of Chemicals (GHS) [7]. Therefore, reliable monitoring of PMZ in aquatic environments is essential. Currently, the detection of promethazine is predominantly performed using conventional analytical techniques such as liquid chromatography–mass spectrometry (LC–MS) [8], enzyme-linked immunosorbent assay (ELISA) [9], and electrochemical sensors [10,11]. LC–MS offers excellent sensitivity and molecular specificity; however, it requires expensive instrumentation, extensive sample pretreatment, and skilled operators, which limits its applicability for rapid on-site or point-of-care analysis [12]. ELISA methods provide relatively high throughput but often suffer from antibody instability, cross-reactivity, and limited shelf life [13]. Electrochemical sensors, while portable and cost-effective, may require complex electrode modification procedures and are susceptible to fouling in complex matrices [14].

Therefore, there remains a need for a detection platform that combines high sensitivity, operational simplicity, real-time monitoring capability, and low cost, particularly for forensic and field-based applications.

Molecularly imprinted polymers (MIPs) are synthetic receptors featuring predesigned recognition cavities that are complementary to target molecules in terms of size, shape, and spatial arrangement of functional groups [15], endowing them with high specificity and affinity toward target analytes. These recognition sites are formed through the polymerization of functional monomers in the presence of template molecules, followed by template removal to generate binding cavities that can selectively rebind the target species [16,17]. Due to their tailor-made selectivity, MIPs have been widely applied in chemical sensing [4], separation [15], and catalysis [18]. MIPs offer several advantages, including superior chemical and thermal stability, lower production cost, ease of preparation [19], and excellent resistance to harsh environmental conditions such as extreme pH, temperature, and organic solvents. These characteristics make MIPs highly attractive as robust and reusable recognition elements for selective sensing applications [20].

Quartz crystal microbalance (QCM)-based sensors are highly sensitive mass-measuring devices that convert interfacial mass variations into real-time frequency shifts based on the piezoelectric properties of quartz crystals [21,22]. Owing to their ability to directly monitor nanogram-level mass changes on electrode surfaces, QCM sensors have been widely recognized as powerful transduction platforms for investigating molecular adsorption and binding processes at solid–liquid interfaces [23]. In addition to their intrinsic high mass sensitivity, QCM sensors offer several advantages, including label-free detection, real-time monitoring capability, simple instrumentation, and ease of integration with functional recognition layers such as molecularly imprinted polymers (MIPs) [24,25]. These features make QCM particularly suitable for the construction of selective biosensing systems [26], where molecular recognition events can be directly translated into quantifiable frequency responses without the need for secondary labeling or amplification steps.

Herein, we report the fabrication of a PMZ-imprinted QCM sensor through regulation of template–monomer interactions during molecular imprinting. Different functional monomer systems were systematically evaluated to optimize intermolecular recognition between PMZ and the polymerizable monomers. In addition, a template protonation strategy was introduced to enhance electrostatic complexation and stabilize the pre-polymerization assembly. By balancing interaction strength and polymerization stability, structurally stable and highly selective imprinting sites were successfully generated.

The resulting MIP-QCM sensor exhibited reliable quantitative recognition toward PMZ together with favorable selectivity, anti-interference capability, and regeneration stability. This work demonstrates that controlled regulation of template-state-dependent intermolecular interactions plays a critical role in improving imprinting-site quality and selective molecular recognition, providing a promising strategy for the imprinting of basic pharmaceutical compounds in complex analytical environments.

## 2. Material and Methods

### 2.1. Reagents and Materials

Promethazine hydrochloride (PMZ, ≥98%), 4-vinylpyridine (4-VP, 95%), 2-acrylamido-2-methylpropanesulfonic acid (AAMPS, 99%), methacrylic acid (MAA, 99%), 2-hydroxyethyl acrylate (HEA, 96%), N,N′-methylenebisacrylamide (MBAA, 99%), ethylene glycol dimethacrylate (EGDMA, 98%), and 2,2′-azobis(2-methylpropionitrile) (AIBN, 12 wt%) were purchased from Sigma-Aldrich(now MilliporeSigma, Burlington, MA, USA). Interfering and matrix compounds including atrazine, pheniramine, pyrimethamine, nicotine, triethylenetetramine (TETA), creatine, 2-nitrophenyl octyl ether, anisaldehyde, oxalic acid, bipyridine, terephthalic acid, and sodium phosphate were obtained from Sigma-Aldrich. Hydrochloric acid (HCl, 37 wt%), sodium hydroxide (NaOH, ≥98%), and nitric acid (HNO_3_, 65 wt%) were also supplied by Sigma-Aldrich. N-phenylenediamine was purchased from Fluka(now part of MilliporeSigma, Burlington, MA, USA), and sodium chloride (NaCl) was obtained from GCE Laboratory Chemicals(GCE Lab Chemicals, Houston, TX, USA). Tap water was collected from the laboratory, and carbonated beverages used for real sample analysis were purchased from a local supermarket. Deionized water was strictly utilized throughout the entire experimental process for solution preparation and sensor rinsing.

### 2.2. Instruments

All aqueous solutions were prepared using ultrapure water (≥18.2 MΩ·cm) produced by a Milli-Q^®^ water purification system (Merck Millipore, Burlington, MA, USA). Quartz crystal microbalance (QCM) measurements were performed using a QCM detection system equipped with gold-coated quartz crystal chips, both of which were supplied by MIPS Innovations Pte Ltd. (Singapore). The surface morphologies of the prepared non-imprinted polymers (NIPs) and molecularly imprinted polymers (MIPs) were characterized by field-emission scanning electron microscopy (FE-SEM). The pH values of the experimental solutions were monitored using a digital pH meter.

### 2.3. Preparation of Molecularly Imprinted Polymers

The molecularly imprinted polymers (MIPs) were synthesized via free-radical precipitation polymerization. In a typical procedure, promethazine hydrochloride (PMZ, 0.0263 mmol, 8.42 mg), functional monomers 4-vinylpyridine (4-VP, 0.21 mmol, 22.08 mg) and 2-acrylamido-2-methylpropanesulfonic acid (AAMPS, 0.21 mmol, 43.52 mg), and the cross-linker ethylene glycol dimethacrylate (EGDMA, 2.1 mmol, 416.26 mg) were dissolved in a mixed solvent system consisting of N,N-dimethylformamide (DMF, 2 mL) and chloroform (8 mL) in a dry 50 mL single-neck round-bottom flask.

A fixed template-to-total-monomer molar ratio of 1:16 was used in all optimized MIP preparations. To regulate the ionization state of promethazine and enhance electrostatic interactions with functional monomers, hydrochloric acid (HCl, 0.2 M) was added at a molar ratio of 1.05 relative to the template. It should be noted that promethazine hydrochloride was used directly as the template without a separate pre-protonation step; the additional HCl was introduced into the polymerization mixture to promote protonation of the tertiary amine group in the organic solvent system (DMF/chloroform), thereby stabilizing electrostatic complexation with the sulfonate groups of AAMPS. In practice, approximately 130 μL of 0.2 M HCl was introduced into the reaction system. A magnetic stir bar was then added, and the solution was purged with high-purity nitrogen for 10 min to remove dissolved oxygen prior to polymerization.

Under continuous nitrogen protection, azobisisobutyronitrile (AIBN, 12 wt% in acetone, 100 µL, corresponding to 0.073 mmol) was injected as the radical initiator, followed by an additional 10 min nitrogen purging. Based on the total vinyl group content (from 4-VP, AAMPS, and EGDMA), the molar ratio of initiator to vinyl groups was approximately 1.58 mol%. The pre-polymerization mixture was subsequently stirred at room temperature for 30 min to allow sufficient interaction between the template and functional monomers.

The polymerization was carried out at 70 °C for 16 h under reflux conditions. After cooling to room temperature, the obtained polymer was collected by vacuum filtration and washed with methanol (3 × 150 mL) to remove unreacted reagents and low-molecular-weight impurities.

The resulting bulk polymer was dried at 60 °C for 12 h and ground into fine powder. Template removal was performed by repeated washing with methanol under stirring (2 h followed by 30 min), until no detectable template remained in the washing solution. Finally, the obtained MIP particles were dried and stored for further use.

The corresponding non-imprinted polymer (NIP) was synthesized under identical conditions as the MIP, with the sole exception that no template (PMZ) was added during the polymerization. The same quantities of functional monomers (4-VP and AAMPS), crosslinker (EGDMA), initiator (AIBN), and solvent system (DMF/chloroform) were used, and the polymerization temperature and duration were kept the same as for the MIP preparation. The resulting NIP was collected, washed, dried, and ground following the same procedure as described for the MIP.

### 2.4. Preparation of MIP-Functionalized QCM Chips

For the fabrication of molecularly imprinted polymer (MIP)-functionalized quartz crystal microbalance (QCM) chips, the synthesized MIP powder was dispersed in n-hexanol (5 mg/mL) and ultrasonicated for 3 min to obtain a homogeneous suspension. Prior to modification, the gold QCM chips were thoroughly cleaned with methanol to remove surface contaminants. In the film deposition process, the primer solution (a polymer solution used to promote adhesion of MIP particles to the gold electrode surface), and the MIP suspension were first mixed at a volume ratio of 1:1 to form a homogeneous coating dispersion. Subsequently, 1.15 μL of the mixed solution was accurately drop-cast onto the central gold electrode of the QCM chip. The modified chip was allowed to stand at room temperature for 1 h, followed by a second deposition of 1.15 μL of the same mixed solution onto the same electrode area. Finally, the chips were left to dry overnight at room temperature to allow complete solvent evaporation and the formation of a uniform and stable MIP recognition film.

### 2.5. Regeneration Procedure

After each measurement cycle, the MIP-modified QCM chip was regenerated by sequential treatment with 1 mmol/L NaOH for 2 min and 10 mmol/L HNO_3_ for 2 min, followed by thorough rinsing with deionized water until the baseline frequency was restored. The rationale for this two-step treatment is as follows: NaOH first disrupts the electrostatic and hydrogen-bonding interactions that bind PMZ to the imprinted cavities via deprotonation; HNO_3_ subsequently neutralizes the alkaline environment and reprotonates the ionizable functional groups, which is expected to help restore the chemical state of the recognition sites. The concentrations (1 mM NaOH and 10 mM HNO_3_) were selected as they provided complete removal of PMZ within 2 min without causing detectable swelling or damage to the polymer film, as verified by stable baseline recovery after each cycle.

### 2.6. Preparation of Real Beverage Samples

To evaluate the practical applicability of the proposed MIP-QCM sensor, commercially available soft drinks (carbonated beverages) were selected as complex matrices for real sample analysis. This matrix contains a variety of potential interferents—including sugars, colorants, acidity regulators, and preservatives—which provide a rigorous test for the anti-interference capability of the sensor. All samples were subjected to a simple and rapid pretreatment procedure. First, the beverages were ultrasonically degassed for 5 min to remove dissolved carbon dioxide. The samples were then filtered using a 0.45 μm polyethersulfone (PES) syringe filter to remove suspended particulate matter and obtain a clear solution. After pretreatment, promethazine standard solutions were spiked into the filtered beverage samples to achieve final concentrations of 100 and 500 ppb (µg L^−1^). Prior to QCM measurement, the pH of each spiked sample was adjusted to 8.5 ± 0.1 using 0.1 M NaOH or 0.1 M HCl to ensure matrix-matched analysis under controlled pH conditions. The samples prepared were then used directly for QCM measurements without further modification.

## 3. Results and Discussion

### 3.1. Optimization of Working pH

To preliminarily determine the optimal working pH for PMZ recognition, the binding response of the NIP toward PMZ (2 ppm) was first investigated over the pH range of 4.0 to 9.5 (Figure S1). As shown in Figure S1, the NIP exhibited a maximum frequency response at pH 8.5, which was therefore selected as the working pH for subsequent experiments. The same pH was also verified to be optimal for the MIP system in separate experiments, and the successful recognition performance of the MIP-QCM sensor (Section 3.3, Section 3.4, Section 3.5, Section 3.6 and Section 3.7) further supported this choice.

### 3.2. Screening of Functional Monomer Systems for PMZ Recognition

**Figure 1:**
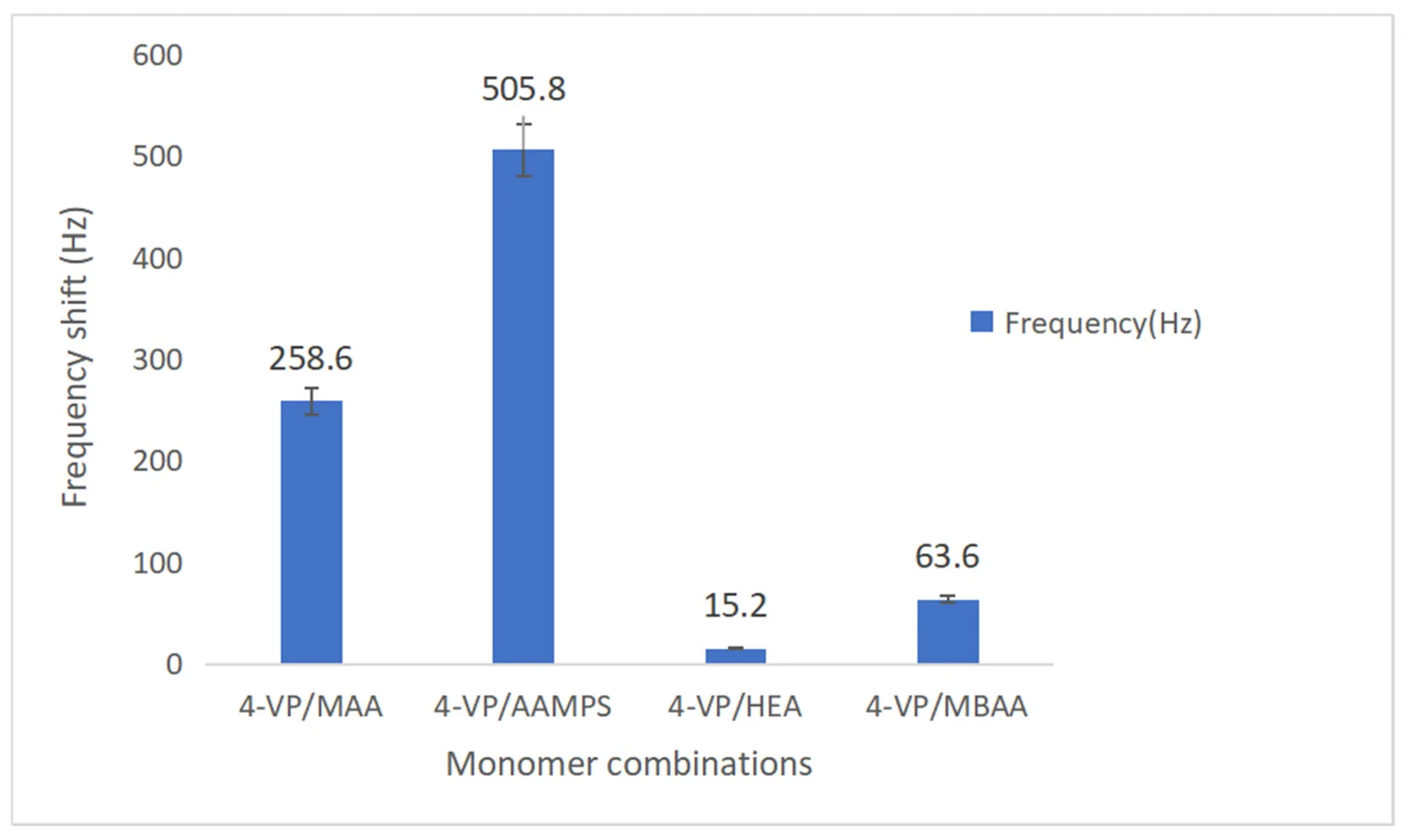
QCM frequency responses of non-imprinted polymers (NIPs) prepared from different 4-VP-based monomer systems toward promethazine (PMZ, 2 ppm) at pH 8.5. Data are presented as mean values of at least three independent measurements (*n* = 3).

To optimize the intermolecular interactions responsible for PMZ imprinting, several 4-VP-based functional monomer systems were investigated using QCM frequency responses under identical conditions, including 4-vinylpyridine (4-VP) combined with methacrylic acid (MAA), 2-hydroxyethyl acrylate (HEA), 2-acrylamido-2-methylpropanesulfonic acid (AAMPS), and N,N′-methylenebisacrylamide (MBAA), as shown in Figure 1. The adsorption behaviors of the corresponding NIPs toward promethazine (PMZ, 2 ppm) were evaluated under pH 8.5 conditions using QCM frequency shift measurements. All data were obtained as the average of at least three independent measurements to ensure reproducibility.

As shown in Figure 1, the frequency responses of the four NIP systems toward PMZ (2 ppm, pH 8.5) were 258.6 Hz (4-VP/MAA), 505.8 Hz (4-VP/AAMPS), 15.2 Hz (4-VP/HEA), and 63.6 Hz (4-VP/MBAA). The 4-VP/AAMPS system exhibited a response approximately 2- to 33-fold higher than the other combinations, indicating significantly stronger template–monomer interactions. This superior performance can be explained by the distinct physicochemical properties of the sulfonate groups in AAMPS. Unlike MAA, which contains relatively weak carboxylic acid groups (–COOH), AAMPS features strongly acidic sulfonic acid groups (–SO_3_H) with a substantially lower pKa, allowing nearly complete deprotonation under pH 8.5 conditions. Consequently, AAMPS predominantly exists as negatively charged sulfonate (–SO_3_^−^), which forms strong and stable electrostatic interactions with protonated promethazine (PMZH^+^, pKa ≈ 9.1). In contrast, the carboxylate groups of MAA, even when partially deprotonated at pH 8.5, provide weaker ionic interactions and are more susceptible to protonation equilibrium fluctuations. Furthermore, the superior ionic hydration stability and higher charge delocalization of sulfonate groups further stabilize the pre-polymerization complex in aqueous environments. Collectively, these factors facilitate strong electrostatic preorganization between protonated PMZ and sulfonate groups, promoting the formation of more homogeneous and stable imprinting cavities. Therefore, the 4-VP/AAMPS system was selected as the preferred functional monomer system for subsequent MIP preparation.

The 4-VP:AAMPS molar ratio was fixed at 1:1 based on equimolar charge balance between the protonated template (PMZH^+^) and the sulfonate groups of AAMPS. While systematic optimization of this ratio could further enhance performance, this composition yielded satisfactory polymerization stability and recognition performance in subsequent experiments and was therefore adopted for all MIP preparations.

### 3.3. Enhancing Electrostatic Complexation via Template Protonation

The systematic optimization of the polymerization conditions is summarized in Table 1. Initially, the absence of polymer formation under neutral conditions (Entry 1) suggests insufficient template–monomer complexation between neutral PMZ and the functional monomers. This phenomenon is mainly attributed to the weak interaction between neutral PMZ and the functional monomers. Since PMZ contains a tertiary amine group and predominantly exists in its neutral form under these conditions, it is difficult to form stable pre-polymerization complexes, thereby hindering the formation of a crosslinked polymer network. In addition, the relatively bulky structure of PMZ may introduce steric hindrance, further disrupting the spatial arrangement of monomers.

To address this issue, hydrochloric acid (HCl) was introduced to protonate PMZ at a carefully controlled molar ratio of 1:1.05 (PMZ:HCl). A direct comparison between Entry 1 (without HCl) and Entry 4 (with HCl) clearly demonstrates that the additional protonation step is essential for successful polymerization and effective imprinting under the current organic solvent system. After protonation, PMZ was converted into the positively charged PMZH^+^, which can form strong electrostatic interactions with the sulfonate groups of AAMPS. The resulting ionic interactions significantly enhanced template–monomer complexation and stabilized the pre-polymerization assembly. Improved molecular organization prior to crosslinking is expected to facilitate the formation of more well-defined imprinting cavities and ultimately enhance the recognition performance of the resulting MIP. These results suggest that regulating the ionization state of the template is an effective strategy for strengthening intermolecular interactions and promoting successful imprinting.

### 3.4. Balancing Interaction Strength and Polymerization Stability

In addition to template protonation, the mass ratio between PMZ and the functional monomers was found to significantly influence the polymerization behavior. As presented in Table 1 (Entries 2–4), different molar ratios (1:4, 1:8, 1:16 and 1:20) were investigated. When the ratio was 1:4 (Entry 2), polymerization could not proceed successfully even after adding HCl, indicating that excessive template loading likely generated overly strong or excessive template–monomer associations, disrupting the formation of a uniform crosslinked network. At a ratio of 1:8 (Entry 3), obvious phase separation was observed, which may be associated with an imbalance in the monomer–template interactions and possible side reactions involving 4-VP. In contrast, the system with a PMZ-to-monomer ratio of 1:16 (Entry 4) exhibited the most stable polymerization behavior and produced polymers with favorable structural integrity. We also investigated a higher ratio of 1:20 (Entry 5). Although polymerization proceeded successfully, the resulting MIP exhibited significantly reduced selectivity toward PMZ compared to the 1:16 system (see Section 3.5). This is likely due to excessive template dilution, which lowered the density of effective imprinting sites and compromised recognition fidelity. These observations indicate that successful imprinting requires a balanced interaction strength: insufficient interactions fail to stabilize the template complex, whereas excessive interactions compromise polymerization stability or recognition performance.

Under these conditions, the polymerization proceeded successfully, yielding structurally stable molecularly imprinted polymers.

These results demonstrate that the ionization state of the template plays a crucial role in the molecular imprinting process. Modulating the protonation state of the template to enhance intermolecular interactions represents an effective strategy for improving polymerization efficiency and imprinting performance, particularly for the imprinting of basic pharmaceutical molecules.

### 3.5. Characterization of the MIP and NIP

The yield of the MIP (79.1%) was slightly lower than that of the NIP (82.2%) based on the mass of recovered polymer relative to the total monomer feed. This difference may be attributed to the presence of the template introducing steric hindrance during polymerization, which partially disrupted the formation of a uniform crosslinked network.

**Figure 2:**
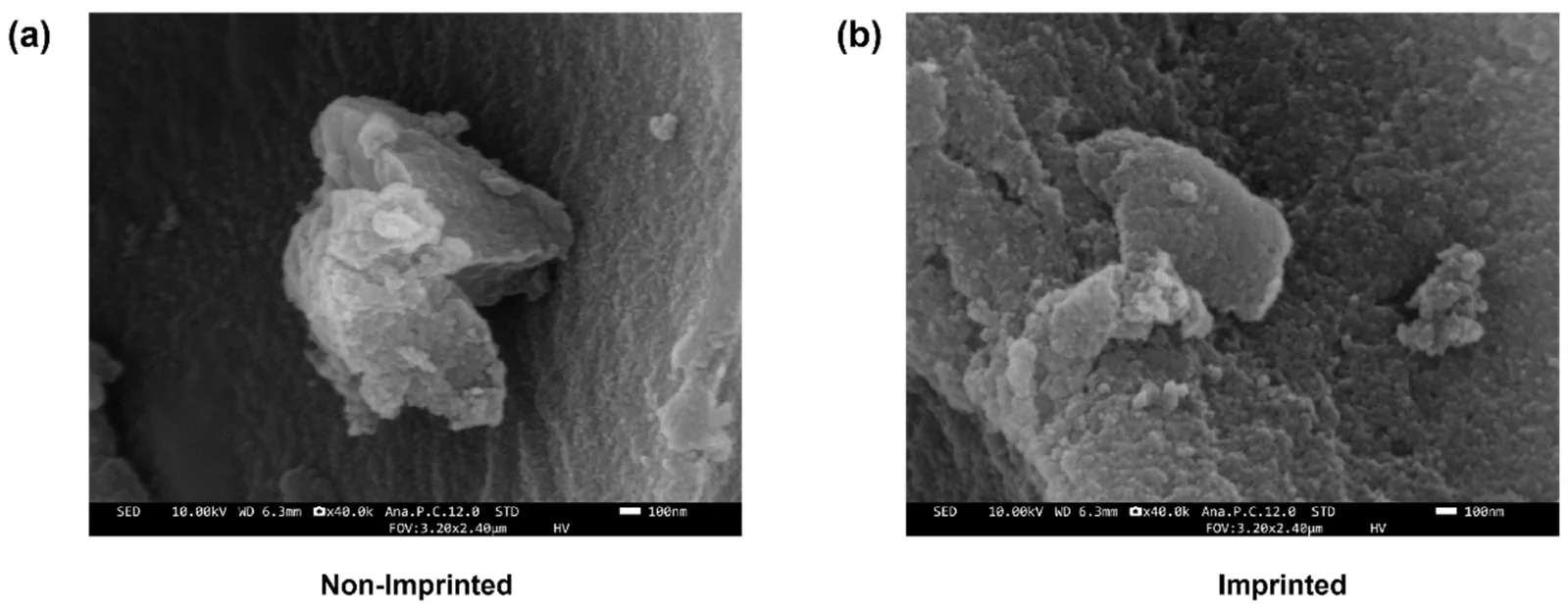
SEM images of (**a**) NIP and (**b**) MIP prepared under the optimized polymerization.

The SEM images reveal noticeable morphological differences between the NIP and MIP materials (Figure 2). The NIP exhibited a relatively compact surface morphology with some surface irregularities. In contrast, the MIP displayed a rougher surface covered with numerous nanoscale granular aggregates. The particle distribution on the MIP surface appeared less uniform and more heterogeneous than that of the NIP, indicating structural changes induced by the imprinting process. The particle sizes of both the MIP and NIP were estimated to be in the range of 2–50 nm, with no significant difference observed between the two materials.

### 3.6. Quantitative Detection Performance

**Figure 3:**
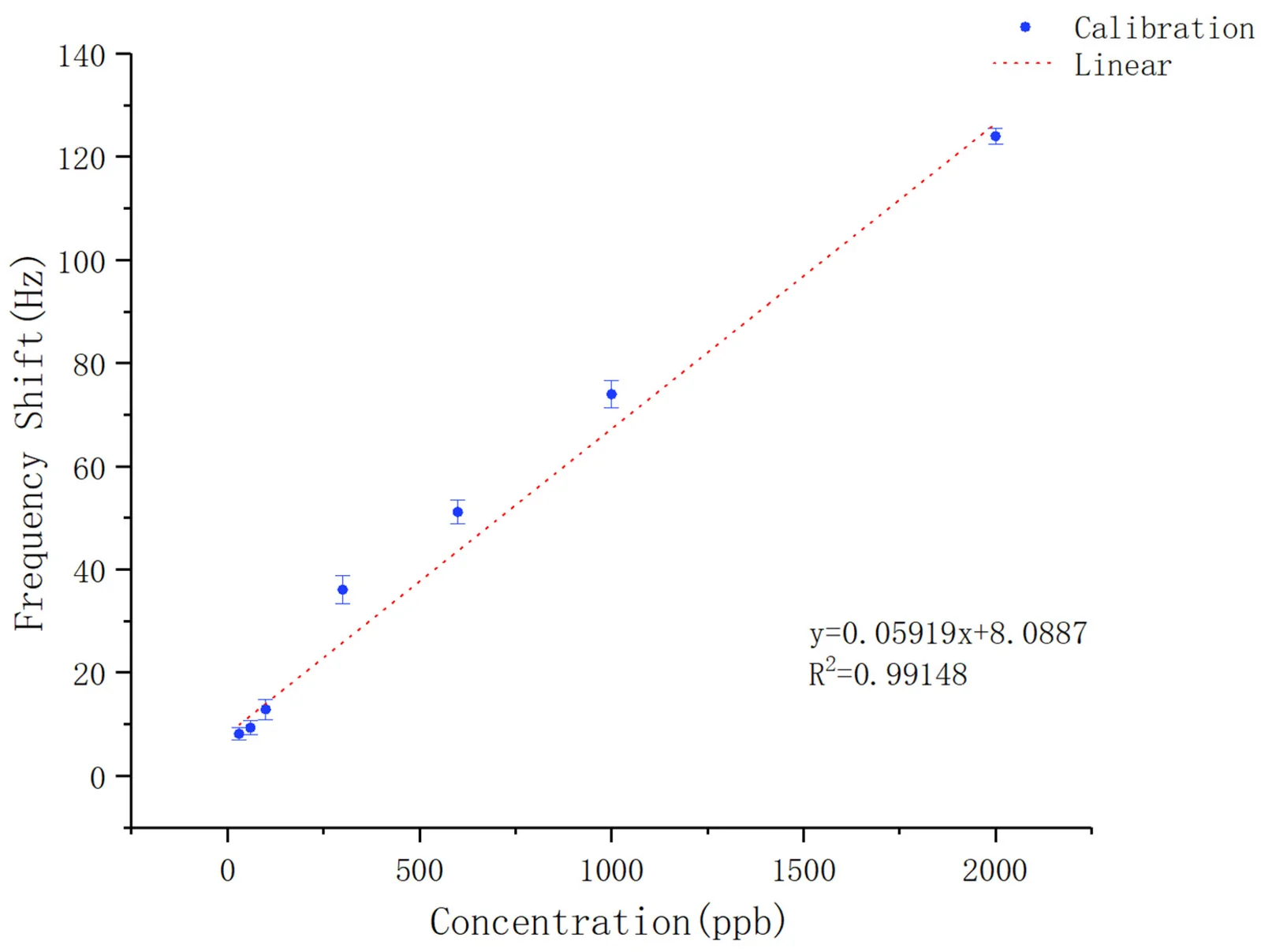
Calibration curve of the MIP-QCM sensor for PMZ detection over the concentration range of 50–2000 ppb under optimized experimental conditions. A satisfactory linear relationship between PMZ concentration and QCM frequency shift was obtained with a correlation coefficient (R^2^) of 0.99148. Error bars represent standard deviations from triplicate measurements (*n* = 3).

The quantitative sensing performance of the prepared MIP-QCM sensor toward PMZ was evaluated over a concentration range of 50–2000 ppb under the optimized experimental conditions (Figure 3). Each data point represents the average of three independent measurements to ensure the reproducibility and reliability of the obtained results. As the PMZ concentration increased, the QCM frequency shift exhibited a gradual and concentration-dependent response, indicating effective adsorption of PMZ molecules onto the imprinted recognition sites of the polymer film.

A satisfactory linear relationship between PMZ concentration and frequency shift was obtained within the investigated concentration range, following the regression equation:

y = 8.0887 + 0.05919x(1)

with a correlation coefficient (R^2^) of 0.99148, demonstrating excellent linearity of the sensing system. The observed concentration-dependent response can be attributed to the increasing occupation of the specific imprinted cavities by PMZ molecules at higher analyte concentrations, resulting in progressive mass loading on the QCM surface.

The good linearity and stable signal response indicate that the prepared MIP-QCM sensor possesses reliable quantitative detection capability toward trace-level PMZ. These results further demonstrate the potential applicability of the sensor for practical pharmaceutical and environmental monitoring analysis.

### 3.7. Analytical Performance

To determine the LOD of the proposed MIP-QCM sensor, the frequency response of seven blank samples (without PMZ) was measured under identical conditions. The standard deviation (σ) of the blank measurements was calculated to be 0.54 Hz. Using the calibration curve slope (*S*) of 0.0582 Hz/ppb, the *LOD* was calculated according to the IUPAC definition LOD=3σ/S. The obtained *LOD* was 27.4 ppb. This result indicates that the sensor is capable of sensitive quantification of promethazine at trace levels.

The imprinting factor (IF) was calculated as the ratio of the net frequency responses of the MIP and NIP toward PMZ (2 ppm) in a tap water matrix, and was determined to be 5.1. This value confirms the effective formation of specific recognition sites in the MIP, consistent with the selectivity and anti-interference results discussed in Section 3.5 and Section 3.6.

### 3.8. Selectivity Analysis

**Figure 4:**
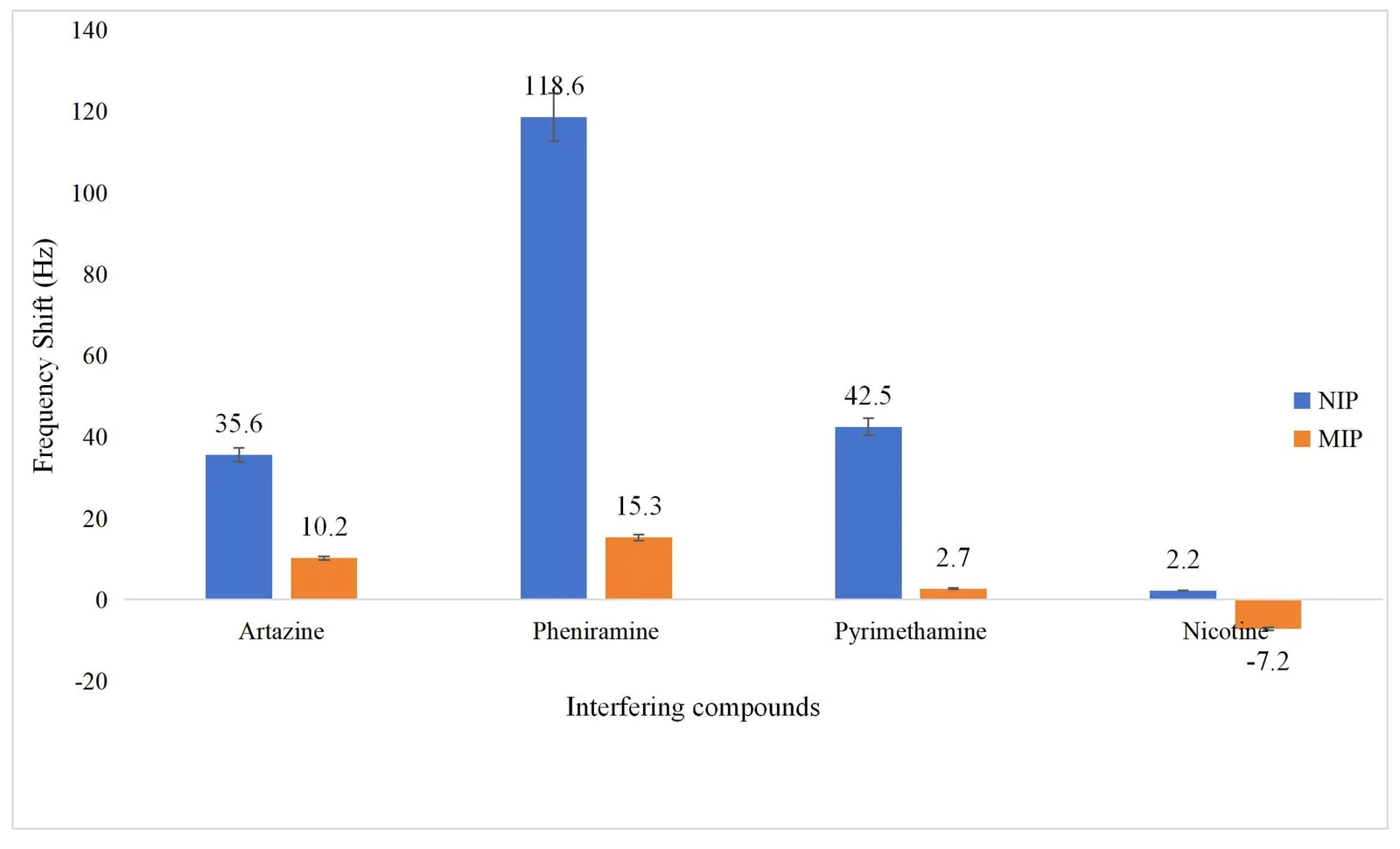
QCM frequency responses of MIP and NIP toward structurally related interfering compounds at pH 8.5 and 2 ppm (*n* = 3).

To investigate the selectivity of the prepared molecularly imprinted polymer (MIP), atrazine, pheniramine, pyrimethamine, and nicotine were employed as interfering analytes, as shown in Figure 4. These compounds were selected due to their structural similarity, aromatic heterocyclic moieties, or potential coexistence in pharmaceutical and environmental matrices. The adsorption behaviors of both the MIP and the corresponding non-imprinted polymer (NIP) toward these compounds were evaluated by QCM frequency shift measurements under pH 8.5 conditions. All analyte solutions were prepared at a concentration of 2 ppm, and the reported results represent the average of at least three independent measurements.

As illustrated in Figure 4, the MIP exhibited relatively low frequency responses toward all interfering compounds, whereas the NIP showed nonspecific adsorption behavior toward the tested analytes. The significantly suppressed responses toward structurally related analogues suggest that the optimized template–monomer interactions contributed to the formation of highly specific recognition cavities for PMZ. In contrast, the MIP prepared at a higher ratio of 1:20 exhibited a substantially higher response toward the interfering analog pheniramine (49.8 Hz at 2 ppm). These results suggest that the prepared MIP possesses favorable molecular recognition capability and good selectivity toward PMZ under the optimized experimental conditions.

### 3.9. Stability and Reusability

The durability of the MIP-QCM sensor was assessed by monitoring its response to 300 ppb PMZ over a 30-day period. Measurements were performed every three days, and each data point represents the average of triplicate independent experiments to ensure statistical reliability. To maintain the sensing performance, a robust regeneration protocol was employed after each detection cycle: the sensor surface was treated with 1 mmol/L NaOH followed by 10 mmol/L HNO_3_ for 2 min each and subsequently rinsed with deionized water until the frequency baseline returned to its initial value.

**Figure 5:**
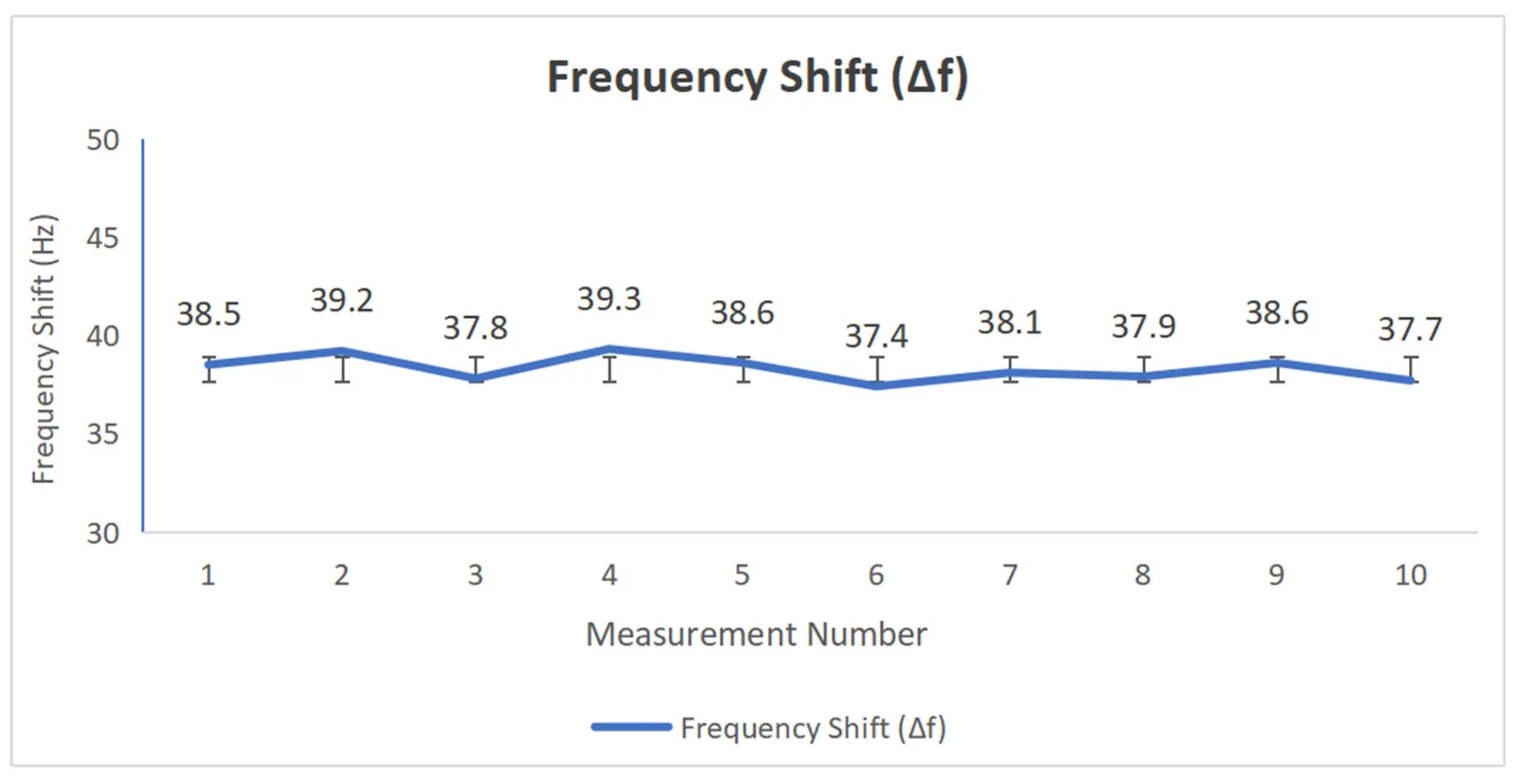
Long-term stability and reusability of the MIP-QCM sensor toward 300 ppb PMZ over 30 days. Measurements were performed every three days. Data are presented as mean ± SD (*n* = 3); error bars represent standard deviations.

As shown in Figure 5, the sensor exhibited exceptional stability, retaining 97.9% of its original sensitivity after 10 regeneration cycles over the one-month period. This indicates that the robust covalent/non-covalent framework of the MIP remained intact during the rigorous acid–base washing cycles. The relative standard deviation (RSD) across all measurements remained below 1.62%, confirming that the imprinted sites and the polymer film possess excellent structural integrity and chemical resistance. This durability highlights the sensor’s potential for practical applications in environmental water monitoring and pharmaceutical residue analysis.

### 3.10. Evaluation of Anti-Interference Performance in Complex Matrices

The anti-interference performance of the prepared MIP-QCM sensor was evaluated under a highly complex chemical environment containing a mixture of potential interfering substances at elevated concentrations (up to 100 ppm for most species), including TETA, creatine, 2-nitrophenyl octyl ether, N-phenylenediamine, NaCl, phosphate, anisaldehyde, oxalic acid, bipyridine, and terephthalic acid, simulating a realistic multi-component interference matrix.

**Figure 6:**
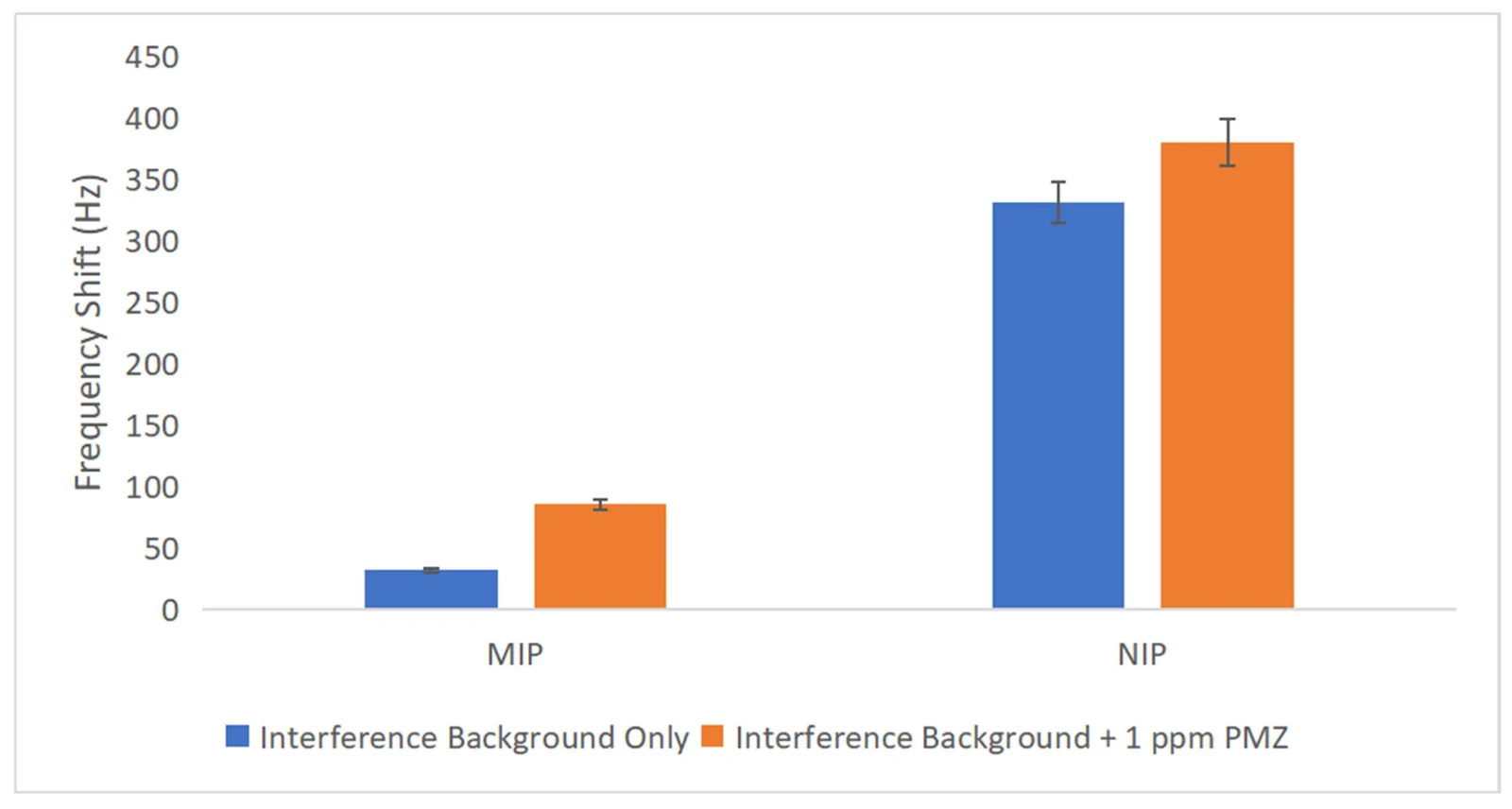
Anti-interference performance of MIP and NIP toward PMZ in a complex matrix containing a mixture of interfering substances (up to 100 ppm). Data are presented as mean ± SD (*n* = 3); error bars represent standard deviations.

As shown in Figure 6, the MIP exhibited a frequency response of only 32.2 Hz toward the interference background alone, indicating limited nonspecific adsorption from the complex matrix. Upon introduction of 1 ppm promethazine (PMZ) into the same interference system, the frequency response increased to 85.7 Hz, demonstrating that the MIP sensor retained its ability to recognize the target molecule even in the presence of high-level competing species. In contrast, the NIP exhibited a significantly higher response (approximately 300 Hz) toward the same interference background, confirming strong nonspecific adsorption in the absence of imprinted recognition sites. These results clearly demonstrate that the imprinting process effectively enhances the selectivity of the sensor by suppressing nonspecific interactions while maintaining target recognition capability under highly competitive conditions.

### 3.11. Analysis of Real Samples

To evaluate the practical applicability of the proposed MIP-QCM sensor for promethazine (PMZ) detection, its analytical performance was tested in real sample matrices, including tap water and carbonated beverages. The samples were spiked with known concentrations of PMZ (100 and 500 ppb), and the recoveries were calculated based on the QCM frequency response calibrated against the standard curve.

As shown in Table 2, the recovery rates ranged from 94.7% to 112.6%, with relative standard deviations (RSD) below 4% (n = 3). Specifically, in tap water, recoveries of 112.6% and 102.7% were obtained for spiked concentrations of 100 and 500 ppb, respectively, with RSD values of 0.54% and 2.68%. In carbonated beverages, recoveries of 94.7% (100 ppb) and 97.2% (500 ppb) were achieved, with RSD values of 2.22% and 3.66%, respectively.

These results demonstrate that the developed MIP-QCM sensors possess satisfactory accuracy and reliability for PMZ detection in complex matrices. The good recovery performance, especially in carbonated drinks containing various additives and sugars, further confirms the sensor’s robust anti-interference capability and its potential for practical applications in pharmaceutical residue monitoring, food safety control, and environmental water analysis.

Table 3 summarizes the analytical performance of the proposed MIP-QCM sensor for promethazine (PMZ) detection in comparison with other reported methods. As shown in Table 3, our sensor achieves a detection limit of 27.4 ppb and a linear detection range of 0.05–2 mg/L. Although the limit of detection is slightly higher than that of the spectrophotometric/PEI-AgNPs method (0.01 ppb), it is significantly lower than those of the colorimetric paper-based spot test (20 ppb), the optical colorimetric sensor (16.5 ppb), and the distance-based paper sensor (33 ppb). More importantly, unlike chromatographic and spectroscopic techniques that rely on expensive and complex instrumentation, our MIP-QCM sensor provides quantitative readout via direct frequency shift measurement using a quartz crystal microbalance, which is cost-effective and easy to operate. Compared to paper-based sensors that require external devices such as cameras or software for image analysis, our sensor eliminates the need for additional equipment, offering a truly user-friendly alternative. Considering its good sensitivity, wide linear range, low cost, and simple operation, the proposed MIP-QCM sensor holds great promise for practical applications in pharmaceutical residue monitoring, food safety control, and environmental analysis.

## 4. Conclusions

A highly selective molecularly imprinted polymer–quartz crystal microbalance (MIP-QCM) sensor was successfully developed for the detection of promethazine through rational regulation of template–monomer interactions during the imprinting process. Systematic investigation of different functional monomer systems revealed that the combination of 4-vinylpyridine and AAMPS provided the most favorable interaction environment for PMZ recognition. More importantly, modulation of the template ionization state through controlled protonation significantly strengthened template–monomer complexation, resulting in improved pre-polymerization organization and the formation of higher-quality imprinting cavities.

The optimized MIP-QCM sensor exhibited a linear detection range of 50–2000 ppb, a detection limit of 27.4 ppb, good selectivity toward PMZ over structurally related compounds, excellent anti-interference capability in complex matrices, and satisfactory regeneration stability. Recoveries of 94.7–112.6% obtained in real sample analysis further demonstrated the practical applicability of the sensor.

Beyond the development of a PMZ sensor, this work provides mechanistic insight into the critical role of template ionization and intermolecular interaction regulation in molecular imprinting. The results suggest that successful imprinting depends not only on monomer selection but also on precise control of the chemical state of the template during pre-polymerization assembly. This strategy may be extended to the imprinting of other basic pharmaceutical compounds and organic amines, offering a general approach for improving imprinting-site fidelity, selectivity and sensing performance.

The findings presented herein provide a useful strategy for the rational design of MIP recognition systems and demonstrate the potential of interaction-engineered MIP-QCM platforms for environmental monitoring, pharmaceutical analysis, food safety assessment, and forensic screening applications.

## Figures and Tables

**Table 1:** Optimization of synthesis conditions for PMZ-MIPs.

Entry	Template State	Molar Ratio (PMZ: Monomers)	HCl Addition (1:1.05)	Polymerization Result
1	Neutral PMZ	1:16	No	No measurable polymer formation
2	Protonated PMZH^+^	1:4	Yes	No measurable polymer formation
3	Protonated PMZH^+^	1:8	Yes	Unstable
4	Protonated PMZH^+^	1:16	Yes	Successful
5	Protonated PMZH^+^	1:20	yes	Successful, poor selectivity

For all entries, the amounts of functional monomers and crosslinker were fixed at 4-VP (0.21 mmol), AAMPS (0.21 mmol), and EGDMA (2.1 mmol), with varying template (PMZ) amounts to achieve the molar ratios listed in the table.

**Table 2:** Recovery results for PMZ detection in real samples using the MIP-QCM sensor (*n* = 3).

Sample Type	Added (ppb)	Found (ppb)	Recovery (%)	RSD (%)
Tap water	100	112.6	112.6	0.54
	500	513.5	102.7	2.68
Carbonated beverages	100	94.7	94.7	2.22
	500	486	97.2	3.66

**Table 3:** Comparison of the proposed MIP-QCM sensor with other reported methods for promethazine (PMZ) detection.

Method	Quantitative Analysis	Limit of Detection (LOD) (mg L^−1^)	Detection Range (mg L^−1^)	Ref.
Colorimetric paper-based spot test	Camera	20	100–600	[27]
Selected ion monitoring method	Gas chromatography and mass spectrometry	0.30	1–10	[28]
Spectrophotometric/PEI-AgNPs method	UV-vis spectrophotometer	0.010	0.03–64.2	[29]
Optical colorimetric sensor	Portable spectrophotometer	16	50–500	[30]
Distance-based paper sensor	Ruler	33	100–700	[31]
MIP-QCM sensor	Quartz Crystal Microbalance	0.027	0.05–2	This work

## Data Availability

The original contributions presented in this study are included in the article and Supplementary Materials. Further inquiries can be directed to the corresponding authors.

## Supplementary Materials

The following supporting information can be downloaded at https://www.mdpi.com/article/10.3390/polym18172146/s1, Figure S1. Effect of pH on the QCM frequency response of NIP toward PMZ (2 ppm) over the pH range of 4.0 to 9.5. Data are presented as mean values of triplicate measurements *n* = 3; error bars represent standard deviations.
