# Supplementary material for: Regulation of Template Ionization and Template–Monomer Interactions for Enhanced Molecular Imprinting and Selective Detection of Promethazine
Source: Polymers (Basel). 2026 Sep 2;18(17):2146. doi: 10.3390/polym18172146 (PMC13568314; doi:10.3390/polym18172146)
Supplement: Supplementary file 1 [file polymers-18-02146-s001.zip › polymers-4502132-supplementary.pdf]

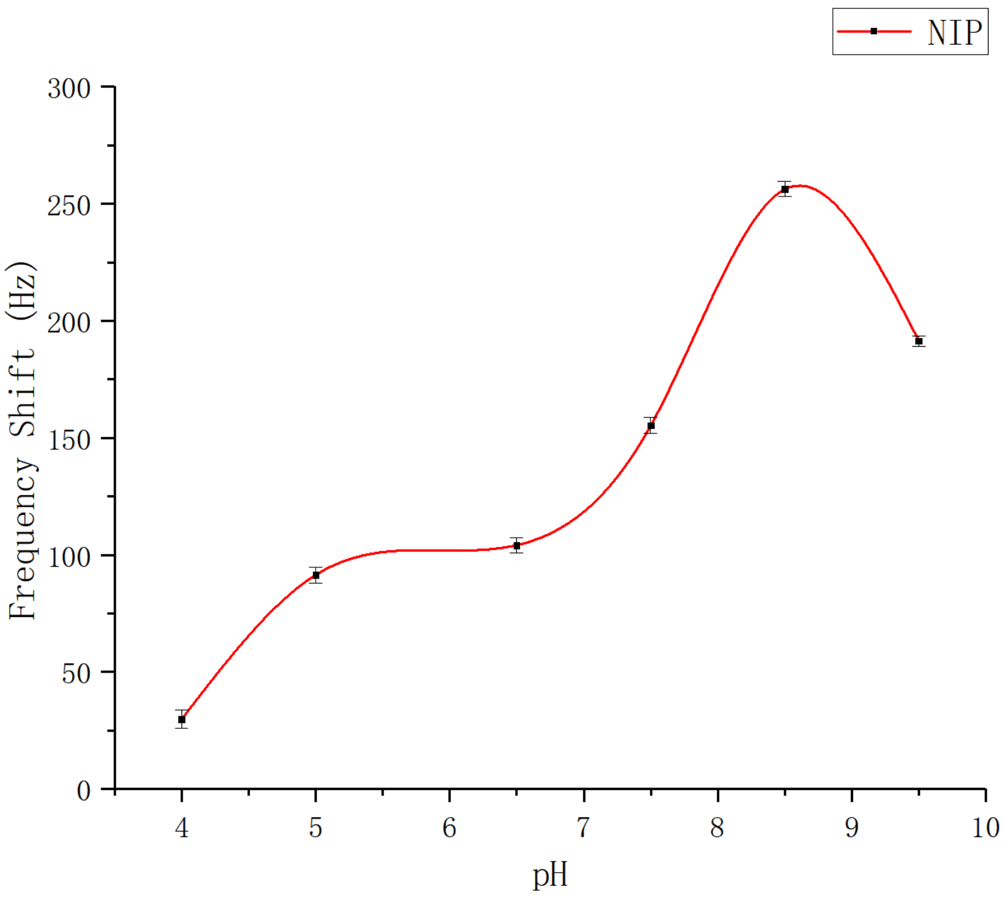

Figure S1. Effect of pH on the QCM frequency response of NIP toward PMZ (2 ppm) over the pH range of 4.0 to 9.5. Data are presented as mean values of triplicate measurements n=3; error bars represent standard deviations.
